# Response of mucinous breast carcinoma to neoadjuvant chemotherapy

**DOI:** 10.55730/1300-0144.5903

**Published:** 2024-05-23

**Authors:** Berkay KILIÇ, Burak İLHAN

**Affiliations:** 1 Department of Surgery, Division of General Surgery, Institute of Oncology, İstanbul University, İstanbul, Turkiye; 2 Department of Surgery, Division of General Surgery, Faculty of Medicine, İstanbul University, İstanbul, Turkiye

**Keywords:** Mucinous breast carcinoma, neoadjuvant chemotherapy, ki-67 index

## Abstract

**Background/aim:**

Mucinous breast carcinoma (MBC) is thought to be a favorable-differentiated form of invasive breast cancer and is rarely preferred for neoadjuvant chemotherapy (NAC). The study aimed to define the response of MBCs to NAC.

**Materials and methods:**

A review was made of the demographic, clinicopathologic characteristics, management and follow-up data of 70 patients diagnosed with MBC between May 2010 and December 2020 by examining the patients’ historical files and oncology records.

**Results:**

The median age, tumor size, and follow-up period of patients were 52.9 (range: 32–87) years, 25.8 (range: 8–88) mm, and 61.5 (range: 18–143) months, respectively. Of the 70 patients, 45 had conservative surgery, 25 had a mastectomy, and 22 had axillary clearance due to a positive sentinel node biopsy or clinical axilla. Eight patients (11.4%) received NAC. Twenty-one patients (30.0%) received adjuvant chemotherapy, whereas almost all the patients received hormone therapy. The preoperative core biopsy diagnosis of four of eight patients receiving NAC was unspecified invasive breast carcinoma. NAC was used as treatment in patients who were younger, had tumors larger in diameter, had tumors with an initial higher T-stage, and especially those with clinically positive axilla, and tumors with a higher Ki-67 index. Despite these preference criteria, both the overall mastectomy and axillary clearance rates were significantly higher in these patients. Two local and five systemic recurrences were observed in the follow-up period. NAC had no significant contribution to survival.

**Conclusion:**

It may be concluded that NAC is not sufficiently effective in either helping to diminish the need for mastectomy by downsizing the tumor, or in preventing axillary clearance in MBCs, and no significant benefit on survival could be observed. In addition, the results may emphasize the importance of identifying the MBC subtype, and the significant association between the degree of response to NAC and the subtype.

## 1. Introduction

Mucinous breast carcinoma (MBC) is a rare type of breast cancer estimated to account for 1%–4% of all breast cancers. MBC is characterized by the presence of a large amount of extracellular mucin and is generally defined as containing more than 50% mucinous components. According to the amount of mucinous content, MBC is further divided into pure MBC (PMBC), which is more frequent, and mixed MBC (MMBC) subtypes. PMBC contains a higher amount of mucinous components than MMBC; more than 90% mucin content (intracellular and extracellular) is defined as PMBC, while less than 90% is defined as MMBC [1]. MBCs usually occur in postmenopausal women and have significantly smaller tumors than more common invasive ductal or lobular variants. A possible explanation for MBCs being diagnosed at smaller sizes could be their slow growth pattern. Furthermore, MBCs have more favorable clinical and pathologic behavior, such as less axillary spread, more frequent estrogen receptor (ER)/progesterone receptor (PR) positivity, and lower rates of human epidermal growth factor receptor 2 (HER2/neu overexpression) [2]. These features reveal that MBC will progress with a more favorable prognosis and survival rates compared to other types of breast cancer [3,4]. In addition, predicting survival is thought to be significantly related to factors including hormone receptor status or adjuvant treatment (AT), as in ductal carcinoma. Hence, for the majority of MBCs, patients undergo surgical removal with additional AT, which often includes antiestrogen therapy. However, most of these factors have not been proven to be significantly prognostic of long-term survival except axillary status. Although nodal positivity is relatively rare, previous studies have shown that node status is the strongest predictor of survival, particularly for MBC [5,6]. Standard management steps for optimal locoregional and systemic control are conducted in line with guidelines similar to ductal carcinoma^1^. Therefore, breast-conserving surgery (BCS) is recommended instead of mastectomy if conditions such as tumor size or tumor/breast volume are appropriate, as the 10- to 15-year local recurrence rates are similar [7]. Axillary staging should also be performed during conservative surgery [8]. Despite all these specific parameters, preoperative approaches have not been clearly validated.

Neoadjuvant chemotherapy (NAC) is often preferred in locally advanced breast cancer that has spread to the axilla. The goals of NAC include reduction of the tumor size to allow conservative surgery, or eliminating axillary metastasis to avoid axillary dissection (AD). Pathological features that may predict better response to NAC are a high tumor grade, HER2/neu overexpression, triple negative immunophenotype, or high Ki-67 proliferation index. It was reported in earlier literature studies that low-grade invasive breast carcinomas, such as MBC, with low proliferation rates do not sufficiently benefit from NAC in terms of survival rates, and are not commonly a candidate for this [9]. This may be attributed to the lower tumor grades, infrequent occurrence of HER2/neu overexpression, or triple negative MBCs. Nevertheless, MBCs with a locally advanced or positive axillary clinical presentation may be thought to necessitate preoperative treatment models. However, clinical experience with NAC for MBC is more limited. Hence, in this study, a single institutional series to define the effects of NAC on MBC.

## 2. Materials and methods

Seventy patients with MBCs were examined and treated between May 2010 and December 2020 at the Oncology Institute, İstanbul University. Carcinomas of unknown stage, T4 or inflammatory carcinomas, cases of unknown treatment status, and those with missing data were excluded. Cases were evaluated in terms of demographics, tumor features, management, and recurrence rates according to historical patient records. The study was approved by the Ethics Committee of İstanbul University (form number: 2024-2356341). All the patients provided written informed consent for participation in the study.

The mass was visualized with ultrasound, mammography, and magnetic resonance imaging when necessary. Usually, core biopsy and less frequently excisional biopsy were used in the preoperative diagnosis. Before NAC or surgery, in the diagnosis, aside from the carcinoma subtype, pathologic parameters including the ER/PR status, HER2/neu status, and Ki-67 proliferation index, if possible, up to the date performed were obtained. Preoperative examinations were usually requested from our pathology department, preferably, but not all examinations were possible. For each hormone receptor status, less than 1% staining by cells was considered as negative. The oncogene human epidermal growth factor receptor 2 (HER2/neu) status was considered negative if the score was 0 or 1+, or positive if the score was 3+. For specimens with a score of 2+, in situ hybridization was used for confirmation of the status. In the definitive examination, macroscopically, MBC can be described as a push-out lesion with a typically gelatinous consistency. Microscopically, MBC has the appearance of groups of malignant cells in mucin pools between thin septa with capillaries. The cell clusters are variable in shape and size with an occasional tubular arrangement. Nuclear atypia is generally low in classic MBC, but in rare cases, atypia and mitoses may prevail [10]. In addition, pathological response in the tumor and axilla is evaluated in patients receiving NAC.

In surgery, wide tumor excision (± reconstruction) was performed for palpable tumors, whereas wire-guided (by ultrasound, mammography) excision was performed for nonpalpable tumors. BCS was performed with a large lumpectomy to achieve a 5 mm margin over the pectoral fascia. The specimens were marked with sutures for orientation in the pathologic examination. Patients with locally advanced tumors, high tumor/breast volume, or multifocal tumors underwent a mastectomy (± reconstruction). Additional mastectomy or reexcision was performed if the margin infiltrated with the ink or invasive or in situ carcinoma was closer than 1 mm at the margin. All the patients underwent a sentinel lymph node biopsy (SLNB). Axillary clearance was performed only if a sentinel lymph node with tumor involvement was detected in the intraoperative examination.

Demographic, clinical, and pathologic data including age, tumor features, receptor status, surgical management, axillary node status, and recurrence rates were collected. Microsoft Excel (Microsoft Luxembourg S.a.r.l., 20 Rue Eugene Ruppert, Luxembourg) was used to record the data. To assess the basic associations of the documented variables, each parameter was tested using the Fisher’s exact test or the chi-squared test in two-tailed univariate analyses. Kaplan–Meier analyses were used to determine the disease-free survival (DFS) and overall survival (OS) rates. p < 0.05 was considered statistically significant. IBM SPSS Statistics for Windows 21.0 (IBM Corp., Armonk, NY, USA) was used in the statistical analyses.

## 3. Results

### 3.1. Study population

The median age, tumor size, and follow-up period of the patients were 52.9 (range: 32–87) years, 25.8 (range: 8–88) mm, and 61.5 (range: 18–143) months, respectively. Thirteen (18.6%) patients had multifocal tumors, and the main tumor of 13 (18.6%) patients was clinical stage three. Sixteen (22.9%) patients were clinically node-positive. In terms of the receptor status, 65 of the tumors (92.9%) were classified as luminal and five (7.1%) were classified as HER2/neu. Of the 70 patients, initially, 48 underwent BCS and 22 underwent mastectomy, whereas after the surgery was changed to mastectomy due to margin positivity were added, 25 had an overall mastectomy. Forty-eight patients had SLNB, and 22 had axillary clearance due to a positive SLNB or clinically positive axilla. Eight patients (11.4%) received NAC. In the NAC protocol, adriamycin + cyclophosphamide (AC) plus taxane were used as the NAC agents for all the patients, and trastuzumab was used for the HER2/neu positive patients. However, neither clinical nor pathological complete response was achieved in any of the patients. Only 21 patients (30.0%) received adjuvant chemotherapy, whereas almost all the patients received hormone therapy. Two local and five systemic recurrences were observed in the follow-up period. The estimated five-year DFS and OS rates were 92.9% and 95.7%, respectively. The preoperative core biopsy diagnosis for four of the eight patients receiving NAC was unspecified invasive breast carcinoma. A general overview of the patients with MBC is shown in Table 1.

### 3.2. Neoadjuvant treatment

Eight of the 70 patients received NAC, whereas the remaining 62 patients received AT. The patients treated with NAC were younger (p = 0.012), had a larger main tumor size (p = 0.008), had tumors with a clinically higher stage (p = 0.004), and had clinically positive axilla (p = 0.001), respectively. Although the total number was small, patients receiving NAC had a higher HER2/neu receptor status compared to those receiving AT (p = 0.009). The Ki-67 scores were higher in the patients receiving NAC (p = 0.001). Despite these preference criteria, both the overall mastectomy (p = 0.02) and axillary clearance (p = 0.001) rates were higher in the patients receiving NAC. Two local and five systemic recurrences were observed in the follow-up period. The frequencies of developing relapse were similar in both patient groups (p = 0.59). The five-year DFS and OS rates were 87.5% vs. 93.5% and 100.0% vs. 96.8% for the patients receiving NAC and AT, respectively. The five-year DFS and OS rates were not statistically different. The patient and tumor characteristics and treatment approaches with NAC and AT are summarized in Table 2.

### 3.3. Survival

**Figure 1:**
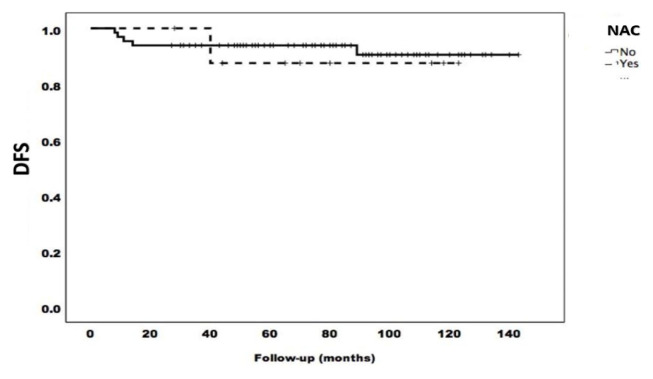
DFS of the patients.

**Figure 2:**
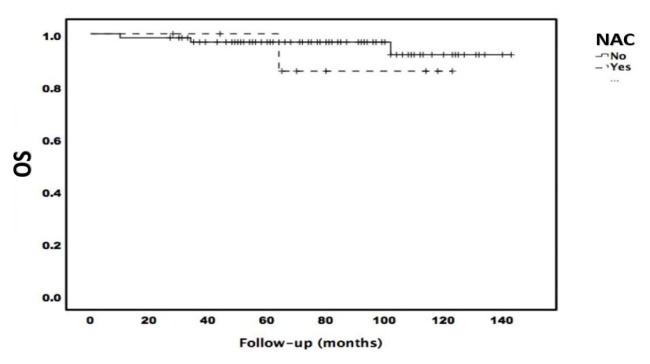
OS of the patients.

There was no difference between the patient groups in terms of the five-year DFS and OS (p > 0.05). Figures 1 and 2 present the survival curves showing the effects of NAC and AT on survival.

## 4. Discussion

MBC is a tumor described by the presence of small roaming clumps of cells in free extracellular pools of mucin. The typical histological features of this tumor have been known for more than 100 years, which suggest that the tumor has a slow progression and good prognosis. In one study, it was reported that prognosis can vary according to the amount of mucin present. MBC has two subtypes; PMBC comprises less than 10% nonmucinous components (previously, mucin-containing mixed histology carcinomas were known as ductal carcinoma with mucinous components or mucinous differentiation), while MMBC comprises 10% or slightly higher nonmucinous invasive components. Both subtypes account for less than 1%–2% of all breast carcinomas and are seen in a wide age group of 15 to 90 years [11]. In addition, it tends to occur at older ages and has a more favorable prognosis than other invasive breast carcinomas. According to the literature, nearly 50% of mucinous carcinomas are diagnosed at >55 years of age, and only 16% of tumors are larger than 5 cm [12,13]. In the current study, 52% of the patients were ≥55 years of age was 52%, and 17% of tumors were larger than 5 cm. Consistent with this, 13 (18.6%) of the main tumors were at the T-stage 3.

Patients are usually diagnosed with screening programs and fewer patients are admitted with a palpable mass. After visualization with routine imaging methods such as mammography, ultrasound, and magnetic resonance imaging, the diagnosis usually follows a core-needle biopsy. The incidence of microcalcification on mammography has been reported as 40% and 20% are occult. Ultrasound usually reveals a round shape with a low frequency of spiculated extensions, but with lobulated contours, encoding minimal blood supply. MBC usually has no axillary lymph node involvement [14]. Park et al. [5] reported a clinical N-positive stage of 21.3%, while in the current study, this rate was 22.9% preoperatively. MBC is typically an ER-positive carcinoma that is treated by antiestrogen therapies in AT [15]. In the literature, it was reported that MBCs have a luminal receptor status in the range of 75%–80%, and 14% have a HER2/neu receptor status. MBCs with HER2/neu receptor status have a good response to trastuzumab as the NAC or AT in general [16]. In our study, the rate of MCs with luminal features was 92.9%, while that with HER2/neu features was 7.1%. Zhou et al. [17] reported that 63.4% of patients with MBCs underwent BCS, whereas the remaining required mastectomy. In another study, it was reported that BCS was sufficient for 69% of MBCs [18]. In the study, of the 70 patients, 45 (64.3%) had overall BCS, 25 (35.7%) had an overall mastectomy. The positive margin rate after BCS requiring conversion to mastectomy was 4.3%. Forty-eight patients had SLNB, and 22 had AD due to a positive SLNB or clinical axilla.

When the survival outcomes were evaluated, it was concluded that MBCs and ductal carcinomas progress differently. Especially for MBCs, the axilla status is decisive in prognosis. As reported by Bae et al. [19], MBC patients had better DFS than invasive ductal carcinoma patients. They also found that AT and the nodal status represented the most significant predictors of prognosis, more than the histological subtype. They reported five-year DFS and OS rates of 95.2%, and 98.9%, respectively. Cao et al. [20] analyzed 309 patients with PMBC and found a five-year DFS and OS of 89% and 95%, respectively. Looking at results reported in the literature, the sample presented by Di Saverio et al. [21] may be regarded as the largest and most relevant. In 11,400 PMBC patients who were retrospectively reviewed, the five-year OS was 94%. The five-year DFS and OS rates were 92.9% and 95.7% in the current study, and a total of seven (10%) relapses were detected, but the prognostic factors related to these patients were not examined.

Several systems can be used to quantify residual disease and/or evaluate the pathological response of breast carcinoma to NAC. One commonly used system is the tumor, node, metastasis (TNM) staging [22]. This system relies on the largest contiguous tumor focus to determine the T stage. It is well recognized that MCB de-novo may show a cellularity of ≤1% [23]. Accordingly, large mucin pools that contain any volume of malignant cells, regardless of cellularity, might be considered for T assessment. Hence, the T stage may remain unchanged from that before therapy, despite a marked reduction in tumor cellularity. Residual cancer burden (RCB) is another system that is frequently used to quantify residual disease after NAC [24]. This system relies on a combination of both cellularity and the tumor bed size. Residual tumors with low cellularity, as was the case for MBCs posttherapy, have a low RCB score. However, in the RCB system, lymph node assessment does not take cellularity into consideration, so the RCB might remain high in patients with low cellular mucin in the lymph nodes. The Miller–Payne system is yet another methodology for assessing chemotherapy response. The system relies fundamentally on a reduction in tumor cellularity compared with the pretreatment cellularity, and this appears to be well suited for tumor response assessment for MBC [25]. However, this system does not take into consideration the response in the lymph nodes, which is an important prognostic factor. Pinder et al. [26] described a system similar to that of Miller–Payne, although it includes quantification of nodal disease, and may be more applicable for our patients; however, it is not widely used at this time. In this retrospective analysis, it was observed that the TNM staging system influenced the preference for NAC, which was significantly more likely to be used in cases with a positive clinical N stage and also preferred for larger tumors with a clinically higher T stage. In addition, NAC was selected for younger patients and those with tumors that had a higher Ki-67 index. NAC has become standard therapy for patients with locally advanced, large, operable breast cancers or with clinically metastatic axillary lymph nodes. This procedure makes BCS possible or provides less AD in the vast majority of breast cancers [16]. However, the use of NAC for MBC is controversial. Some studies have shown that they benefit from NAC with appropriate receptor status, but others have shown no contribution, and therefore, patients with locally advanced tumors are good candidates for primary surgery according to these studies [25].

This study had some serious limitations. The study may extrapolate that the use of NAC is not quite effective in preventing axillary clearance, but this finding cannot be generalizable. In addition, NAC was chosen for patients with large tumors, and clinically more advanced stage tumors to control the margin status or reduce the number of reexcisions/mastectomies, but there was a significant discordance with the high rates of mastectomies performed in the patients receiving NAC. However, due to the small number of the total patients, and small number of patients receiving NAC, no general determination about this could be made.

In conclusion, the study revealed that patients with MBC have a significant likelihood of undergoing mastectomy and axillary clearance. NAC was used in clinically axilla-positive tumors to avoid axillary clearance, and in larger and clinically more advanced tumors to avoid mastectomy. However, it was highlighted that NAC did not offer a valuable contribution to reducing the axillary metastasis and mastectomy rates. In addition, NAC was chosen for tumors with HER2/neu receptor status, and a high Ki-67 proliferation index, but did not confer any significant survival advantage, even in tumors with these tumor characteristics. In summary, NAC cannot provide a sufficiently effective response in MBC patients. Moreover, it is important to identify the subtype of invasive breast carcinoma, as it may cause the degree of response to NAC to vary.

## Figures and Tables

**Table 1:** Total outcomes of the patients (n = 70) with MBC.

**Age**	**Median (range)**	52.9 (32–87)
**Main tumor, size, mm**	**Median (range)**	25.8 (8–88)
**Follow-up period (months)**		61.5 (18–146)
**Multifocal**	**n**	**%**
No	57	81.4
Yes	13	18.6
**cT stage**		
T 1–2	57	81.4
T 3	13	18.6
**cN**		
Negative	54	77.1
Positive	16	22.9
**Receptor status**		
Luminal	65	92.9
HER2/neu	5	7.1
**NAC**	8	11.4
**Initial surgery**		
BCS	48	68.6
Mastectomy	22	31.4
**Margin positivity**	6	8.6
**Overall surgery**		
BCS	45	64.3
Mastectomy	25	35.7
**Axilla**		
SLNB	48	68.6
AD	22	31.4
**Relapse**	7	10.0
**Five-year DFS**	92.9%	
**Five-year OS**	95.7%	

BCS: breast-conserving surgery, SLNB: sentinel lymph node biopsy, AD: axillary dissection

**Table 2:** Differences between patients receiving NAC and AT.

		NAC (n = 8)	AT (n = 62)	p
**Age**		44.4 (±4.8)	54.0 (±10.4)	0.012
**Main tumor, size, mm**		39.5 (±17.9)	24.0 (±14.8)	0.008
**Multifocal**	No	7 (87.5%)	50 (80.6%)	0.54
	Yes	1 (12.5%)	12 (19.4%)	
**cT stage**	1–2	3 (37.5%)	54 (87.1%)	0.004
	3	5 (62.5%)	8 (12.9%)	
**cN**	Negative	2 (25.0%)	52 (84.9%)	0.001
	Positive	6 (75.0%)	10 (16.1%)	
**Receptor status**	Luminal	5 (62.5%)	60 (96.8%)	0.009
	HER2/neu	3 (37.5%)	2 (3.2%)	
**Ki-67**		9.7 (±3.9)	30.6 (±12.4)	0.001
**Initial surgery**	BCS	3 (37.5%)	45 (72.6%)	0.06
	Mastectomy	5 (67.5%)	17 (27.4%)	
**Margin positivity**		1 (12.5%)	5 (8.1%)	0.53
**Overall surgery**	BCS	2 (25.0%)	43 (69.4%)	0.02
	Mastectomy	6 (75.0%)	19 (30.6%)	
**Axilla**	SLNB	1 (12.5%)	50 (80.6%)	0.001
	AD	7 (87.5%)	12 (19.4%)	
**Relapse**	No	7 (87.5%)	56 (90.3%)	0.59
	Yes	1 (12.5%)	6 (9.7%)	
**Five-year DFS**		87.5%	93.5%	0.47
**Five-year OS**		100.0%	96.8%	0.78

NAC: neoadjuvant chemotherapy, AT: adjuvant treatment.

## Footnotes

1 The National Comprehensive Cancer Network guidelines of treatment of cancer by site: breast cancer. Ver. 1.2023 [Online]. Website http://www.nccn.org/professionals/physician_gls/pdf/breast.pdf [accessed 19 September 2023].
